# Oral and Pancreatobiliary Microbiota in Cancer: A Systematic Review of Compositional Alterations and Their Clinical Implications

**DOI:** 10.1111/jop.70172

**Published:** 2026-07-20

**Authors:** Esperouz Fariba, Villani Rosanna, Ciavarella Domenico, Serviddio Gaetano, Lorusso Mauro, Lo Russo Lucio

**Affiliations:** ^1^ Department of Clinical and Experimental Medicine, School of Dentistry University of Foggia Foggia Italy; ^2^ Department of Medical and Surgical Sciences, Institute of Internal Medicine, C.U.R.E. (University Centre for Liver Disease Research and Treatment) University of Foggia Foggia Italy; ^3^ Department of Medical and Surgical Sciences University of Foggia Foggia Italy

**Keywords:** 16 rRNA gene sequencing, oral microbiota, pancreatobiliary cancers

## Abstract

**Background:**

Associations between microbial dysbiosis and malignancies of the pancreatobiliary system have been described in recent studies. As a result of the limited number of studies that have been done specifically on the malignancies of the biliary tract, information regarding oral, biliary and tumour‐related microbial alterations was combined to provide an overview of the microbial changes that may occur.

**Methods:**

Studies that involved observations on the composition or presence of dysbiosis of the microbiota from oral (saliva, oral rinse, dental plaque) or other non‐oral specimens (bile, pancreatic tissue, duodenal tissue and bacteria‐derived extracellular vesicles from plasma) in patients with pancreatobiliary malignancies were considered. The risk of bias was evaluated using the Newcastle‐Ottawa Scale (NOS) for case–control study designs and the JBI Checklist for cross‐sectional studies.

**Results:**

There were a total of 16 studies involving 1426 participants that were conducted using both case–control and cross‐sectional study designs. Samples were collected from saliva, oral wash, bile, pancreatic and duodenal tissues and bacterial extracellular vesicles isolated from plasma samples. The most common method used was 16S rRNA sequencing, and two used shotgun metagenomics. In all the studies, patients with pancreatobiliary cancers, especially PDAC, had a significantly higher abundance of opportunistic microbes like Streptococcus, Veillonella, Fusobacterium, Prevotella and a lower abundance of commensal bacteria like Neisseria and Corynebacterium. There was overlap of microbial profile in the oral cavity and tumour/bile compartments in a few studies.

**Conclusions:**

Although the current data is preliminary and observational in nature, there are consistent findings linking microbiota dysbiosis with cancers of the pancreatobiliary region within both oral and non‐oral body sites. Causality has not been established yet. The use of microbial signatures as a basis for biomarker development is a promising research direction.

## 1 Introduction

The oral microbiota is a complex and dynamic ecosystem consisting of over 700 bacterial species that play a fundamental role in maintaining homeostasis in the oral cavity and, more generally, systemic health [1]. In recent years, it has emerged that alterations in microbial composition, known as dysbiosis, can contribute not only to local diseases such as periodontitis, but also to chronic systemic conditions, including various types of neoplasms [2].

Oral dysbiosis differs from simple microbiota variation in that it presents a pathological imbalance characterised by reduced microbial diversity, enrichment of pathobionts, depletion of beneficial commensals, functional disruption of metabolic and immune pathways and altered host–microbe interactions [3]. While microbiota variation may reflect physiological adaptations on environmental or dietary changes, dysbiosis implies a sustained perturbation of the oral ecosystem with downstream inflammatory and immunomodulatory consequences [3]. It is through these dysbiotic mechanisms, rather than mere compositional differences, that the oral microbiome may hypothetically contribute to carcinogenesis via chronic inflammatory signalling, production of carcinogenic metabolites and systemic immune dysregulation [2].

Biliary tract cancers (BTCs), which include intrahepatic and extrahepatic cholangiocarcinoma, gallbladder carcinoma and tumours of the ampulla of Vater, represent a heterogeneous group of neoplasms characterised by poor prognosis and often late diagnosis [4]. Despite diagnostic and therapeutic advances, the global incidence of BTC is increasing and long‐term survival remains limited [5]. In this context, the identification of new early biomarkers and potential pathogenic targets is a research priority.

Several recent studies have suggested a possible link between oral microbiota and biliary tract tumours [6, 7, 8, 9, 10]. It has been observed that the oral bacterial composition differs significantly in patients with BTC compared to healthy controls or those with benign diseases, with variations in alpha and beta diversity and specific modifications of certain bacterial taxa [11].

In particular, Akkermansia was found exclusively in the bile of patients with BTC [12], while Corynebacterium and Streptococcus showed altered patterns in the different study groups [13]. Other studies have shown that microbial profiles associated with plasma extracellular vesicles can accurately discriminate between subjects with BTC and controls, suggesting a potential diagnostic value [14, 15, 16]. The biological mechanisms underlying this association remain largely hypothetical. It has been proposed that oral dysbiosis may promote chronic inflammatory and systemic immunomodulatory processes, contributing to a pro‐tumour microenvironment in the pancreaticobiliary tract [17]. Furthermore, factors such as bacterial translocation, activation of pathogen‐associated molecular patterns (PAMPs) and increased circulating inflammatory mediators (e.g., C‐reactive protein) may mediate the link between oral health and biliary carcinogenesis [18].

Although pancreatic ductal adenocarcinoma, cholangiocarcinoma, gallbladder carcinoma and periampullary tumours are distinct oncological entities with important differences in anatomy, carcinogenesis and inflammatory microenvironment, they share several biological features that justify their joint consideration within a common oral‐pancreatobiliary framework [19]. First, all these malignancies arise from ductal or glandular epithelia exposed to bile and pancreatic secretions, environments in which microbial colonisation and dysbiosis may exert analogous pro‐inflammatory and carcinogenic effects [20]. *Second, they share common risk factors including chronic biliary inflammation, primary sclerosing cholangitis, choledocholithiasis and metabolic syndrome, conditions that have been independently associated with oral dysbiosis* [6]. Third, the anatomical contiguity of the pancreatobiliary tract and its shared embryological origin from the foregut suggest that microbial translocation and immune modulation occurring at one site may affect the entire district [21].

Overall, current evidence [6] suggests a potentially significant association between alterations in the oral microbiota and BTCs, although causality remains to be proven. This systematic review aims to summarise and critically appraise the current evidence examining the relationship between the oral microbiota and pancreatobiliary malignancies, including pancreatic ductal adenocarcinoma and periampullary cancers. Given the limited number of studies specifically focused on biliary tract neoplasms, the available evidence from pancreatic and related pancreatobiliary tumours was integrated to provide a comprehensive overview of potential biological mechanisms, microbial signatures and their implications for early detection and clinical outcomes.

## 2 Review

The current systematic review was conducted in accordance with the Preferred Reporting Items for Systematic Reviews and Meta‐analyses (PRISMA) standards. The protocol was filed and registered in the PROSPERO database CRD42024581512.

### 2.1 Search Strategy and Database Screening

An electronic search of the English‐language literature up to October 2025 was conducted using the following databases: PUBMED, SCOPUS and Web of Science. A direct search was also conducted in the bibliographies of all reviewed articles. The research was carried out utilising a combination of mesh terms and free text phrases, together with some Boolean operations (AND, OR). The protocol for PUBMED was as follows: (“Oral Microbiota” OR “Oral microbiome” OR “Salivary microbiota” OR “Salivary microbiome” OR “Oral bacteria” OR “Saliva microbiota” OR “Oral dysbiosis” OR “Periodontal microbiota” OR “Subgingival microbiota” OR “Oral Microbiota”) AND (“Biliary Tract Neoplasms” OR “Cholangiocarcinoma” OR “Gallbladder Neoplasms” OR “Pancreatic Neoplasms” OR “biliary tract cancer” OR “biliary cancer” OR “pancreatobiliary cancer” OR “pancreatic ductal adenocarcinoma” OR “PDAC” OR “periampullary cancer” OR “ampullary tumor” OR “extracellular vesicles” OR “bacterial extracellular vesicles”).

The same search strategy was used for Scopus and Web of science. Search strategies for each specific database are available in supplemental materials.

### 2.2 Eligibility Criteria

Studies were included if they met the following criteria. First, only English‐language publications were considered as this is the primary language of scientific communication in biomedical research and ensure consistency in data extraction and interpretation. Second, only observational study designs were eligible, including cohort, case‐control, cross‐sectional, retrospective and longitudinal studies since the research question concerns an association between microbial exposure and cancer. Third, studies had to be conducted exclusively on adult participants (> 18 years old), given that pancreaticobiliary malignancies are predominantly adult‐onset diseases and that the oral microbiome undergoes substantial maturation changes during adolescence. Additionally, studies analysing non‐oral sample sources, including bile, pancreatic tissue, duodenal tissue and plasma‐derived bacterial extracellular vesicles, were also considered eligible when they provided complementary evidence of microbial overlap with oral taxa, with the aim of exploring the plausibility of an oral–pancreatobiliary microbial continuum.

Studies were excluded if they were systematic reviews or meta‐analyses. Studies not in English were excluded due to the risk of translation bias and inconsistency in data extraction (Reasons for exclusion of full‐text articles are provided in Table S1).

### 2.3 Focused PICO Question and Effect Measure

*(P) Participants:* Adults (≥ 18 years) diagnosed with pancreatobiliary malignancies, including pancreatic ductal adenocarcinoma, distal cholangiocarcinoma or ampullary cancers.

*(I) Interventions:* Determination of the microbiome composition, diversity or dysbiosis by: (1) oral specimens such as saliva, unstimulated whole saliva, oral wash, dental plaque and subgingival plaque; or (2) additional non‐oral specimens such as plasma‐derived bacterial extracellular vesicles, bile, pancreas and duodenum, where there was evidence of the similarity of bacteria found in the oral cavity to those found in other tissues.

*(C) Comparison of Interventions:* Microbial diversity and taxonomic profiles compared with healthy individuals or with participants without pancreatobiliary malignancies.

*(O) Outcome measures:* Differences in alpha and beta diversity; identification of specific oral taxa associated with pancreatobiliary cancers; and exploration of potential diagnostic or prognostic roles of oral microbial profiles.

### 2.4 Studies Screening and Inclusion

Two authors (FE and RV) independently screened the obtained citations by reading the titles and abstracts of them. They developed a screening list of full‐text eligibility after exclusion and inclusion criteria assessment. In case of discrepancy, they referred to the third author (LLR) and made a final decision.

### 2.5 Data Extraction

Similarly, two reviewers (FE and RV) independently extracted data, and the findings were compared and consolidated using an ad hoc extraction sheet. Articles where there was disagreement were re‐screened in a consensus meeting with the third reviewer (LLR). The Excel extraction sheet contained the following fields: author, country, study design, sample size, cancer type, taxa outcomes (Table 1).

**TABLE 1:** General characteristics of the included studies.

Studies	Countriess	Tumour types	Bacterial taxa	Direction of changes	Direction of changes	Sample sources	Sequencings/Methods	Main findings
Segura‐López et al. (2015)	Mexico	BTC (extrahepatic CCA)	*Helicobacter bilis*	*H. bilis* ↑ versus non‐cancer controls (bile)	Associated with extrahepatic cholangiocarcinoma	Bile	16S rRNA	*H. bilis* linked to infection‐related carcinogenesis
Maruyama et al. (2017)	Japan	BTC (bile duct cancer)	Enterococcus, Fusobacterium	*Enterococcus*, *Fusobacterium* ↑ versus healthy controls (bile)	Increased in BTC	Bile	16S rRNA	Enrichment of Enterococcus associated with bile duct obstruction
Fan et al. (2018)	USA	PDAC	*Porphyromonas gingivalis* , *Aggregatibacter actinomycetemcomitans*	*P. gingivalis* , *A. actinomycetemcomitans* ↑ versus controls; *Leptotrichia* ↓ versus controls	Increased in PDAC; Leptotrichia ↓	Oral wash	16S rRNA	Prospective nested case–control; *P. gingivalis* associated with increased PDAC risk (OR 1.60)
Wei et al. (2020)	China	PDAC	Streptococcus, Leptotrichia	*Streptococcus*, *Leptotrichia* ↑ versus HC; *Veillonella*, *Neisseria* ↓ versus HC	Increased in PDAC; Veillonella, Neisseria ↓	Saliva	16S rRNA	Salivary microbiome distinguishes PDAC from healthy controls
Sun et al. (2020)	China	PDAC	*Fusobacterium periodonticum*	*Fusobacterium periodonticum* ↑ versus HC; *Neisseria mucosa* ↓ versus HC	Increased in PDAC; *Neisseria mucosa* ↓	Saliva	16S rRNA	*Fusobacterium periodonticum* and *Neisseria mucosa* as potential diagnostic biomarkers
Lee H. et al. (2020)	South Korea	BTC	Corynebacterium, Comamonas, Pseudomonadaceae	*Corynebacterium*, *Comamonas* ↓ versus HC (plasma EVs)	Decreased in BTC	Plasma (bacterial EVs)	16S rRNA	Predictive microbial EV signature for BTC
Söder et al. (2021)	Finland	Mixed malignancy	*Aggregatibacter actinomycetemcomitans*	*A. actinomycetemcomitans* ↑ versus HC	Increased in malignancy	Saliva	16S rRNA	Association between oral pathogens and cancer
Okuda et al. (2022)	Japan	BTC	Akkermansia	*Akkermansia* ↑ versus non‐BTC controls (bile)	Present only in BTC bile	Bile	16S rRNA	Linked to altered bile microbiota and external drainage
Narayanan et al. (2023)	United Kingdom	BTC	Streptococcus, Veillonella	*Streptococcus*, *Veillonella* ↑ versus HC	Increased in BTC	Saliva, subgingival plaque	16S rRNA	Enrichment correlated with IL‐6 and CRP
Nouri et al. (2023)	United Kingdom	BTC	Prevotella, Streptococcus	*Prevotella*, *Streptococcus* ↑ versus HC	Enriched in cancer	Saliva	16S rRNA	Distinct oral microbiome profile in BTC
Chen et al. (2023)	China	PDAC	Granulicatella, Veillonella, Peptostreptococcus, Alloprevotella	*Granulicatella*, *Veillonella*, *Peptostreptococcus*, *Alloprevotella* ↑ versus HC (saliva); *Prevotella*, *Coprobacter* ↑ versus HC (faeces)	Increased in PDAC	Saliva + faeces	16S rRNA	Oral microbiome diagnostic model AUC 0.916 for PDAC
McKinley et al. (2023)	USA	PDAC	*Veillonella atypica* , Streptococcus	*V. atypica* , *Streptococcus* high prevalence in both saliva and tumour tissue (no HC comparator)	High prevalence in saliva and PDAC tumour tissue	Saliva + pancreatic tissue	16S rRNA + rrn + metagenomics	Oral‐to‐tumour translocation of *V. atypica* confirmed by sequence similarity
Shibata et al. (2024)	Japan	BTC	Oribacterium, Stomatobaculum	*Oribacterium*, *Stomatobaculum* ↑ versus non‐tumour controls	Increased in tumour group	Saliva, duodenum	16S rRNA	Higher abundance in tumour mucosa of biliary cancer patients
Padure et al. (2025)	Romania	PDAC + BTC	Streptococcus, Gemella, Granulicatella, Neisseria, Veillonella	*Streptococcus*, *Gemella*, *Granulicatella* ↑ post‐chemotherapy versus pre‐chemotherapy; *Neisseria* ↓	Streptococcus ↑ post‐chemotherapy; Neisseria ↓	Saliva	16S rRNA	Chemotherapy‐induced microbial shifts
Uguz et al. (2025)	Turkey	PDAC + BTC	Streptococcus, Clostridium, *Rothia mucilaginosa* , *Veillonella dispar*	*Streptococcus*, *V. dispar* , *R. mucilaginosa* ↑ versus HC; *C. perfringens* ↑ in BTC versus PDAC	Increased in pancreatic and biliary cancers	Saliva, pancreatic tissue	16S rRNA	Shared oral–tumour microbiota; *C. perfringens* enriched in BTC
Oh et al. (2025)	South Korea	Upper GI cancers (incl. PDAC)	Akkermansia, Escherichia–Shigella, Subdoligranulum	*Akkermansia*, *Escherichia–Shigella*, *Subdoligranulum* ↓ versus HC	Decreased in upper GI cancers	Saliva	Metagenomics	Diagnostic model for gastrointestinal malignancies

Abbreviations: BTC = biliary tract cancer; PDAC = pancreatic ductal adenocarcinoma.

### 2.6 Assessment of Risk of Bias

The Newcastle–Ottawa Scale (NOS) was applied to the 11 case–control studies [22, 23, 24, 25, 26, 27, 28, 29, 30, 31, 32], while the JBI Critical Appraisal Checklist for Analytical Cross‐Sectional Studies was used for the five cross‐sectional studies [33, 34, 35, 36, 37]. The assessments were performed independently by two reviewers (FE and RV) and conflicts were resolved in a consensus session with the third reviewer (LLR).

## 3 Results

### 3.1 Study Selection

The search retrieved a total of 279 records, from electronic databases (100 from PubMed and 179 from Web of Science). After removal of 86 duplicate records, 193 articles were screened by title and abstract, of which 182 were excluded as out of scope, 11 proceeded to full‐text assessment and 5 were newly included. An additional 12 studies were identified through citation searching and bibliography screening. The final review thus includes 16 studies in total [22, 23, 24, 25, 26, 27, 28, 29, 30, 31, 32, 33, 34, 35, 36, 37] (Figure 1).

**FIGURE 1:**
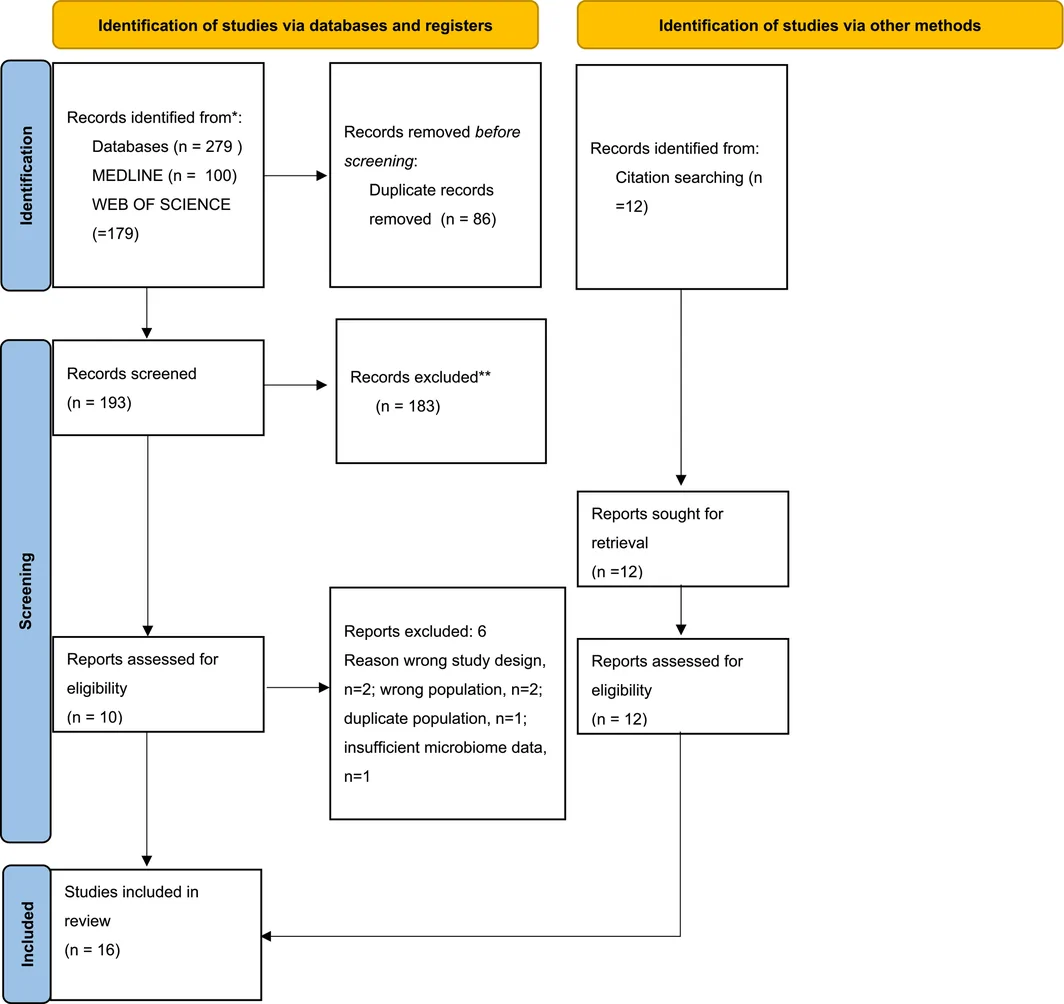
Flowchart of studies' selection process.

### 3.2 Study Characteristics

A total of 16 studies [22, 23, 24, 25, 26, 27, 28, 29, 30, 31, 32, 33, 34, 35, 36, 37] published between 2015 and 2025, were included, encompassing 1426 participants from Japan [28, 30, 36], South Korea [25, 29], Finland [37], Romania [35], Mexico [31], the United Kingdom [26, 27], Turkey [32], the United states [22, 23] and China [24, 33, 34]. All studies were observational, mainly case–control or cross‐sectional.

### 3.3 Pancreatic Ductal Adenocarcinoma Studies (Oral/Salivary Sampling)

Six studies focused specifically on PDAC using oral or salivary sampling [22, 23, 24, 32, 33, 34]. Fan et al. [23] conducted the methodologically most robust study in the PDAC subset, a population‐based prospective nested case–control study comprising 361 incident pancreatic adenocarcinoma cases and 371 matched controls from two large US cohorts, with pre‐diagnostic oral wash samples collected up to 10 years before cancer diagnosis, substantially reducing the risk of reverse causation. Chen et al. [24] performed a multisite analysis of both oral and gut microbiota in 94 participants, constructing a random forest diagnostic model based on salivary microbiome data that achieved an AUC of 0.916 for discriminating PDAC from healthy controls.

### 3.4 Biliary Tract Cancer Studies (Oral, Bile and Plasma Sampling)

Seven studies addressed BTC, including cholangiocarcinoma, gallbladder carcinoma and ampullary tumours, using saliva [26, 27, 37], bile [27, 28, 31] or plasma‐derived bacterial extracellular vesicles [29].

### 3.5 Studies Demonstrating Oral–Tumour Tissue Overlap

Two studies [22, 32] provided direct evidence of microbial continuity between the oral cavity and tumour tissue: McKinley et al. (PDAC) and Uguz et al. (mixed pancreatic/biliary), supporting the biological plausibility of an oral–pancreatobiliary axis.

These sample sources represent biologically distinct compartments and are not considered equivalent evidence of oral microbiota involvement; findings are therefore interpreted according to sample origin throughout the synthesis.

### 3.6 Risk of Bias

Among case–control studies [22, 23, 24, 25, 26, 27, 28, 29, 30, 31, 32], two studies [23, 25] achieved the maximum NOS score of 9/9, reflecting rigorous participant selection, adequate confounder control and validated exposure assessment. Six further studies [22, 24, 27, 28, 29, 32] scored 7–8, indicating low risk of bias. The remaining three [26, 30, 31] scored 5–6, corresponding to moderate risk, primarily due to limited confounder adjustment and smaller sample sizes (Figure 2). Among cross‐sectional studies assessed with the JBI checklist, Shibata et al. [36] met all eight criteria (Score 8/8, low risk). Wei et al. [33] and Padure et al. [35] scored 7/8 and 6/8, respectively (low‐to‐moderate risk), with partial concerns regarding confounder management. Sun et al. [34] scored 5/8 (moderate risk), mainly owing to the small sample size and heterogeneous benign disease comparator group. Söder et al. [37] scored 4/8 (high risk), as the study setting was insufficiently described and no strategy for confounder control was reported (Figure 3). Risk of bias assessments using the Newcastle–Ottawa Scale and the JBI Critical Appraisal Checklist for cross‐sectional studies are provided in Tables S2 and S3, respectively.

**FIGURE 2:**
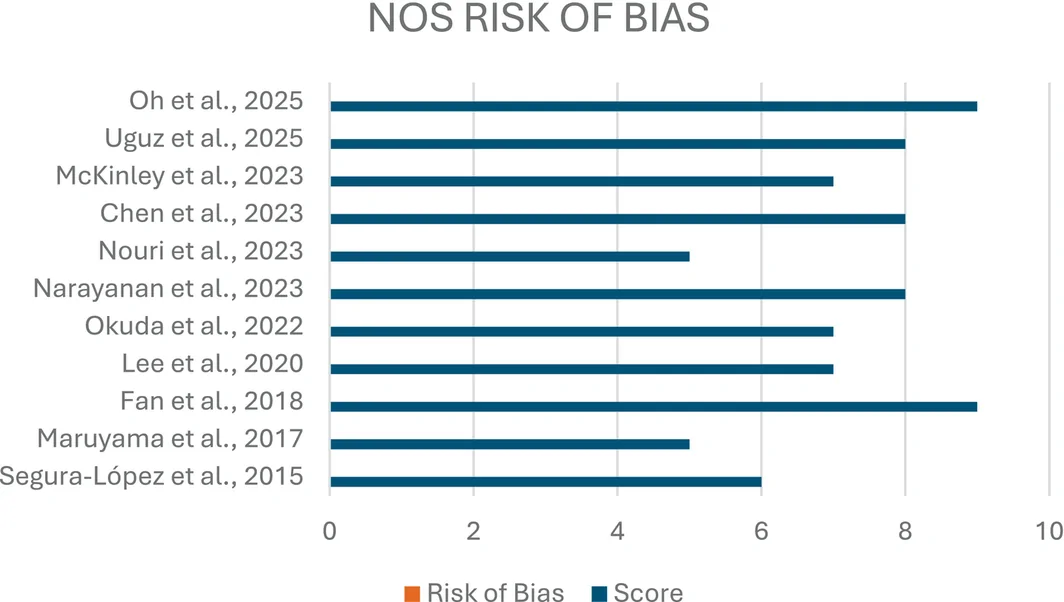
Study quality as assessed by the Newcastle Ottawa Scale (NOS).

**FIGURE 3:**
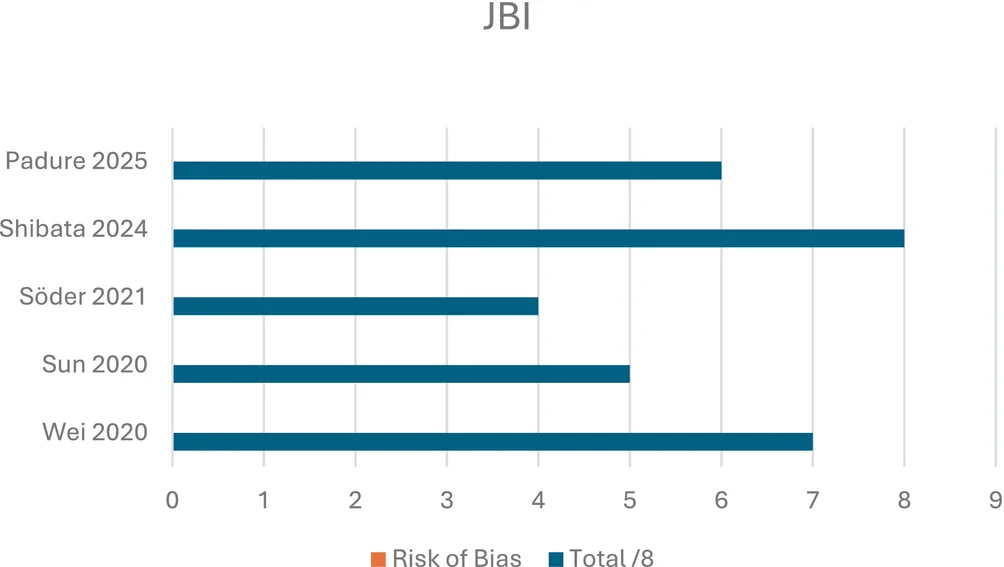
Study quality as assessed by the JBI.

### 3.7 Synthesis of Findings and Certainty of Evidence

Considering the significant clinical and methodological heterogeneity of the studies included in this paper, which included different tumour types, sample types, sequencing methods and comparison populations, a meta‐analysis with pooled effect sizes was not possible. Findings will thus be presented in a structured narrative synthesis stratified by tumour type and sample type (Table 2). Where appropriate, quantitative measures of association were presented for each individual study, such as odds ratios, area under the receiver operating characteristic curve (AUC), linear discriminant analysis (LDA) score and *p* value for differential abundance (Table 2). The certainty of evidence was evaluated using the GRADE system. In all cases, the certainty of evidence for primary associations described in this review was deemed to be LOW. Downgrading was done due to the following reasons: observational study design and thus potential reverse causation bias (−1); imprecision due to small sample size in most studies (−1); inconsistent taxonomic findings between studies and across tumour types (−1); and indirectness because most mechanisms proposed are based on evidence from experiments conducted in other gastrointestinal cancer studies (−1). There are no upgrading criteria.

**TABLE 2:** Structured narrative synthesis of results: Effect sizes, association measures, and GRADE certainty of evidence.

Studies	Cancer types	Key taxa/outcomes	Measures	Values	Comparators	GRADE certainty
Segura‐López et al. (2015)	BTC (eCCA)	*H. bilis* ↑	OR	2.83 (95% CI 1.49–5.32)	Versus non‐cancer controls (bile)	LOW
Maruyama et al. (2017)	BTC (bile duct)	*Enterococcus*, *Fusobacterium* ↑	*p*	*p* = 0.033 (periodontitis–survival)	Versus HC (bile)	LOW
Fan et al. (2018)	PDAC	*P. gingivalis* ↑ *A. actinomycetemcomitans* ↑ *Leptotrichia* ↓	OR OR OR	1.60 (95% CI 1.15–2.22) 2.20 (95% CI 1.16–4.18) 0.87 (95% CI 0.79–0.95)	Versus matched controls (oral wash, prospective)	LOW
Wei et al. (2020)	PDAC	*Streptococcus* ↑ *Leptotrichia* ↑ *Veillonella* ↓ *Neisseria* ↓	OR OR OR OR	5.34 (95% CI 1.28–22.28) 6.89 (95% CI 1.42–33.34) 0.19 (95% CI 0.06–0.63) 0.31 (95% CI 0.10–0.95)	Versus HC (saliva)	LOW
Sun et al. (2020)	PDAC	*Fusobacterium periodonticum* ↑ *Neisseria mucosa* ↓	LDA score (LEfSe)	log10 LDA > 4.0 log10 LDA > 4.0	Versus HC and BPD (saliva)	LOW
Lee H. et al. (2020)	BTC	EV microbial signature (5‐marker panel)	AUC	1.000 (95% CI 0.852–1.000)	Versus HC (plasma EVs)	LOW
Söder et al. (2021)	Mixed malignancy	*A. actinomycetemcomitans* ↑	OR	1.25 (95% CI 1.03–1.52)	Versus non‐cancer controls (saliva)	LOW
Okuda et al. (2022)	BTC	*Akkermansia* ↑ (bile‐specific)	*p*	*p* = 0.041	Versus non‐BTC controls (bile)	LOW
Narayanan et al. (2023)	BTC	*Streptococcus*, *Veillonella* ↑ (saliva + subgingival)	*p* value (differential abundance)	*p* < 0.05 (exact values NR)	Versus HC	LOW
Nouri et al. (2023)	BTC	ML classifier (*Prevotella*, *Streptococcus* ↑)	AUC F2 score	AUC = 0.98 F2 = 0.96	Versus HC (saliva)	LOW
Chen et al. (2023)	PDAC	Oral diagnostic model (*Granulicatella*, *Veillonella* ↑) Gut diagnostic model (*Prevotella*, *Coprobacter* ↑)	AUC AUC	0.916 (95% CI 0.832–1.000) 0.856 (95% CI 0.74–0.972)	Versus HC (saliva + faeces)	LOW
McKinley et al. (2023)	PDAC	*V. atypica* , *Streptococcus* (saliva + tumour tissue)	Sequence similarity	99% (rrn operon) saliva vs. tumour	No HC comparator	LOW
Shibata et al. (2024)	BTC	*Oribacterium*, *Stomatobaculum* ↑ (saliva + duodenum)	*p* value (differential abundance)	*p* < 0.05 (exact values NR)	Versus non‐tumour controls	LOW
Padure et al. (2025)	PDAC + BTC	*Streptococcus*, *Gemella*, *Granulicatella* ↑ *Neisseria* ↓	*p* value (diversity + abundance)	Shannon *p* = 0.0009 Simpson *p* = 0.0001	Post‐ versus pre‐chemotherapy (saliva)	LOW
Uguz et al. (2025)	PDAC + BTC	*Streptococcus*, *Veillonella*, *Rothia mucilaginosa* ↑	*p* value	*p* < 0.05–0.0001	Versus HC (saliva + pancreatic tissue)	LOW
Oh et al. (2025)	Upper GI (incl. PDAC)	Metagenomic classifier (*Akkermansia*, *E.‐Shigella*, *Subdoligranulum* ↓)	AUC	0.791 (PDAC vs. HC)	Versus HC (saliva, metagenomics)	LOW

*Note:* GRADE certainty of evidence: The overall certainty of evidence for the association between oral/pancreatobiliary microbiota dysbiosis and pancreatobiliary malignancies is rated LOW across all included studies. Downgrading factors include: (1) observational study design (cross‐sectional or case–control) with inherent risk of reverse causation; (2) small sample sizes in most studies, reducing precision; (3) substantial inconsistency in taxonomic findings across studies and tumour types; (4) indirectness—most mechanistic inferences are derived from experimental data in other gastrointestinal cancers; (5) moderate risk of bias in several studies due to inadequate confounder control. No upgrading factors were identified. These limitations preclude pooled quantitative synthesis; findings are presented as a structured narrative synthesis.

Abbreviations: AUC = area under the curve; BPD = benign pancreatic disease; BTC = biliary tract cancer; CI = confidence interval; eCCA = extrahepatic cholangiocarcinoma; EVs = extracellular vesicles; HC = healthy controls; LDA = linear discriminant analysis; ML = machine learning; NR = not reported; OR = odds ratio; PDAC = pancreatic ductal adenocarcinoma.

## 4 Discussion

This systematic review synthesises evidence from 16 observational studies investigating the relationship between oral and biliary microbiota and pancreatobiliary malignancies. Despite substantial heterogeneity in study population, design, sample source and sequencing methodology, findings are broadly consistent in suggesting an association between oral microbial dysbiosis and these cancers, though the strength and specificity of this association varies by tumour type and sample compartment. Across the included studies, opportunistic oral taxa such as Streptococcus, Veillonella, Fusobacterium and Prevotella were observed at higher relative abundance in BTC and PDAC patients compared to healthy controls in several studies [27, 28, 31, 35, 36, 37], while commensal genera such as Neisseria and Corynebacterium were depleted [26, 27, 32, 35] These patterns are broadly consistent with dysbiotic signatures reported in other gastrointestinal malignancies, where certain oral‐derived species have been proposed, largely on the basis of experimental data, to contribute to tumourigenesis through immune evasion, cytokine activation and DNA damage; however, direct mechanistic evidence in BTC is lacking [26, 27, 28, 36]. Some studies have reported overlapping bacterial communities in saliva and bile [28], providing preliminary observational support for the concept of an oral–biliary microbial continuum, though the directionality and functional significance of this overlap remain to be established. Several authors have hypothesised that oral microbes could translocate along the gastrointestinal tract or via the haematogenous route, potentially colonising biliary epithelial cells and contributing to chronic inflammation [28, 31, 32]. It has been further speculated, largely on the basis of experimental and gastrointestinal cancer data rather than direct evidence from the included studies, that such microbes might influence bile acid metabolism, contribute to carcinogenic secondary bile acid production, and modulate the tumour microenvironment through toll‐like receptor signalling and NF‐κB–mediated pathways [26, 27, 28, 31, 32]. These mechanisms remain speculative in the context of pancreatobiliary cancers and await direct experimental validation. Streptococcus and Veillonella, facultative anaerobes involved in lactate metabolism, have been hypothesised to thrive in hypoxic or inflamed microenvironments and to potentially exacerbate local immune activation, though this has not been directly demonstrated in biliary tissue [26, 27, 28, 35]. Fusobacterium species have been shown, primarily in colorectal cancer models, to produce adhesins (FadA, Fap2) that may facilitate epithelial adhesion and inflammatory cytokine secretion; whether comparable mechanisms operate in biliary carcinogenesis remains unknown [26, 27, 31]. Such activities have been proposed, primarily in experimental models of gastrointestinal carcinogenesis, to sustain a pro‐tumoural environment by recruiting myeloid‐derived suppressor cells (MDSCs) and polarising tumour‐associated macrophages (TAMs); whether analogous processes occur in biliary carcinogenesis remains to be established [26, 27, 31]. Beyond putative local mechanisms, the systemic dimension of oral dysbiosis deserves consideration. Narayanan et al. [27] reported elevated circulating levels of IL‐6, CRP and TNF‐α in BTC patients with altered oral microbiomes, suggesting that oral dysbiosis may be associated with systemic inflammatory activation, although the cross‐sectional design of this study precludes causal inference. It is equally plausible that elevated systemic inflammation in cancer patients secondarily alters the oral ecosystem, rather than the reverse. These observations are consistent with the broader concept of a gut–liver–oral axis, a proposed bidirectional communication system wherein oral microbial communities may interact with hepatic and biliary systems through immune and metabolic signalling pathways [38]. However, it is important to emphasise that these mechanistic links are largely inferred from the broader gastrointestinal microbiome literature and from experimental models, rather than directly demonstrated within the included studies themselves, nearly all of which are observational and cross‐sectional in design. The discovery of microbial extracellular vesicles (EVs) in plasma [29] represents a potentially non‐invasive avenue for biomarker discovery. EVs carry bacterial DNA, proteins and metabolites reflective of the host–microbiota interface and, in preliminary analyses, have shown capacity to discriminate BTC from benign biliary disease or healthy controls [29]. These findings are exploratory and require prospective validation before diagnostic utility can be established. From a clinical perspective, the present findings are hypothesis‐generating rather than practise‐changing. They suggest that periodontal health and oral microbial balance warrant investigation as potential modifiable factors in pancreatobiliary oncogenesis, but clinical translation requires prospective evidence of causality that does not yet exist. Patients with chronic periodontitis, primary sclerosing cholangitis or recurrent cholangitis might represent populations of interest for future microbiome‐monitoring studies. Interventions such as periodontal treatment, dietary modulation, probiotics or prebiotics should be regarded as putative strategies pending confirmation of causal relationships [39]. Taken together, these findings are consistent with the emerging view that the oral microbiome is not an isolated ecosystem but may participate in broader systemic and gastrointestinal processes. Should future prospective studies confirm causal or predictive relationships, integration of oral microbiome profiling into hepatobiliary oncology could open avenues for early diagnosis, risk stratification and targeted prevention. At present, however, these possibilities remain exploratory.

## 5 Limitations

Even though there is mounting evidence, some limitations should be mentioned. The restriction to English‐language publications represents a potential source of language bias, as relevant studies published in other languages may have been missed.

Most of the included studies were cross‐sectional and therefore did not permit temporal or causal relationships between oral dysbiosis and BTC onset. Where sample sizes were small, in most instances, they reduced statistical power and increased the risk of Type II errors. Heterogeneity in sequencing methods (different 16S rRNA variable regions, coverage depth and bioinformatic pipelines) also prevents comparability of microbial profiles between studies.

Again, another key point to consider is the geographical clustering of studies, because the majority of studies were based in East Asia, in which lifestyle food intakes and genetic backgrounds could be different from those in other groups. Most of the confounding factors such as exposure to antibiotics, smoking, alcohol consumption, periodontal disease and metabolic diseases were not well controlled. There are no longitudinal studies or functional tests available, which restricts the ability to clarify whether microbial changes are causes or consequences of carcinogenesis.

Future research needs to be aimed at large‐scale, prospective, multicentre studies integrating shotgun metagenomics, metabolomics and host immune profiling to better delineate the causal role and clinical significance of the oral microbiome in BTC.

## 6 Conclusions

This systematic review finds consistent, though non‐uniform, links between dysbiotic changes in oral microflora and cancers of the pancreas and biliary tree. While some microorganisms (*Streptococcus, Veillonella and Fusobacterium species*) were more abundant in cancer patients' samples, other taxa (Neisseria) were typically less abundant, but the exact microbial signature was dependent on tumour type (PDAC vs. BTC), sample material (saliva, bile, tumour tissue, plasma EVs) and patient population, thereby precluding any definitive generalisations. The causative nature is unknown yet, so the existing evidence base should be considered hypothesis generating. The findings nevertheless indicate that oral microbiome profiling may become a promising approach to biomarker identification and further research in pancreatobiliary oncology. Further large‐scale prospective multicentre studies employing shotgun metagenomics, metabolomics and immune profile analysis with proper adjustment for potential confounders and longitudinal follow‐up will be necessary to determine whether oral microbial dysbiosis is causally linked to PDAC/BTC and what implications may arise from it for risk stratification/diagnosis/treatment.

## Author Contributions

Conceptualisation: Esperouz Fariba, Lo Russo Lucio. Data curation and formal analysis: Lorusso Mauro. Funding acquisition: Serviddio Gaetano. Investigation methodology: Ciavarella Domenico. Project administration: Lo Russo Lucio and Serviddio Gaetano. Supervision: Villani Rosanna. Validation: Villani Rosanna. Visualisation writing – original draft and writing – review and editing: Esperouz Fariba.

## Funding

The authors have nothing to report.

## Conflicts of Interest

The authors declare no conflicts of interest.

## Supporting information


**Table S1:** Studies excluded at full‐text assessment with reasons for exclusion.

**Table S2:** Risk of bias assessment—Newcastle–Ottawa Scale (NOS).

**Table S3:** Risk of bias assessment—JBI critical appraisal checklist for cross‐sectional studies.

**Data S1:** PRISMA 2020 checklist.

## Data Availability

The data that support the findings of this study are available from the corresponding author upon reasonable request.
