# Supplementary material for: Oral and Pancreatobiliary Microbiota in Cancer: A Systematic Review of Compositional Alterations and Their Clinical Implications
Source: J Oral Pathol Med. 2026 Jul 20;55(9):1008–18. doi: 10.1111/jop.70172 (PMC13635271; doi:10.1111/jop.70172)
Supplement: Supplementary file 1 [file JOP-55-1008-s001.docx]

**Table S1. Studies excluded at full-text assessment with reasons for exclusion**

*In accordance with PRISMA 2020 item 16b, the following table lists all studies assessed at full-text stage that were subsequently excluded, with the specific reason for exclusion for each.*

| **#** | **Author** | **Year** | **Title** | **Reason for exclusion** |
| --- | --- | --- | --- | --- |
| 1 | Mauceri R. | 2023 | No Clear Clustering Dysbiosis from Salivary Microbiota Analysis by Long Sequencing Reads in Patients Affected by Oral Squamous Cell Carcinoma: A Single Center Study | Wrong population: oral squamous cell carcinoma, not a pancreatobiliary malignancy |
| 2 | Zhang Y. | 2025 | Oral microbiota and biliary tract cancers: unveiling hidden mechanistic links | Wrong study design: narrative review, not an original observational study |
| 3 | Rao B.C. | 2022 | Alterations in the human oral microbiome in cholangiocarcinoma | Duplicate population: overlapping cohort already captured via citation searching |
| 4 | Arteta A.A. | 2022 | Cholangiocyte derived carcinomas and local microbiota | Wrong exposure: does not assess oral or pancreatobiliary microbiota composition |
| 5 | Mima K. | 2021 | The microbiome, genetics, and gastrointestinal neoplasms: the evolving field of molecular pathological epidemiology | Wrong study design: review article, not an original observational study |
| 6 | Nouri Z. | 2023 | Exploring Connections between Oral Microbiota, Short-Chain Fatty Acids, and Specific Cancer Types | Wrong population: focuses on oral, head and neck, and gastric cancer, not pancreatobiliary malignancies |
