# Supplementary material for: Oral and Pancreatobiliary Microbiota in Cancer: A Systematic Review of Compositional Alterations and Their Clinical Implications
Source: J Oral Pathol Med. 2026 Jul 20;55(9):1008–18. doi: 10.1111/jop.70172 (PMC13635271; doi:10.1111/jop.70172)
Supplement: Supplementary file 2 [file JOP-55-1008-s002.docx]

**Table S3. Risk of Bias Assessment – JBI Critical Appraisal Checklist for Cross-Sectional Studies**

*Applied to cross-sectional studies (n = 5)*

| **JBI Criterion** | **Wei 2020** | **Sun 2020** | **Söder 2021** | **Shibata 2024** | **Padure 2025** |
| --- | --- | --- | --- | --- | --- |
| 1. Inclusion criteria defined | ✅ | ✅ | ✅ | ✅ | ✅ |
| 2. Subjects & setting described | ✅ | ✅ | ⚠️ | ✅ | ✅ |
| 3. Exposure measured validly | ✅ | ✅ | ✅ | ✅ | ✅ |
| 4. Objective criteria for condition | ✅ | ⚠️ | ⚠️ | ✅ | ✅ |
| 5. Confounders identified | ✅ | ⚠️ | ❌ | ✅ | ⚠️ |
| 6. Strategies to manage confounders | ⚠️ | ❌ | ❌ | ✅ | ❌ |
| 7. Outcomes measured validly | ✅ | ✅ | ✅ | ✅ | ✅ |
| 8. Appropriate statistical analysis | ✅ | ✅ | ✅ | ✅ | ✅ |
| **Total /8** | **7** | **5** | **4** | **8** | **6** |
| **Risk of Bias** | **Low** | **Moderate** | **High** | **Low** | **Moderate** |

*✅ = Yes (criterion met)	⚠️ = Unclear / partially met	❌ = No (criterion not met)*

*Score interpretation: 7–8 = Low risk; 5–6 = Moderate risk; ≤4 = High risk.*
