# Supplementary material for: Oral and Pancreatobiliary Microbiota in Cancer: A Systematic Review of Compositional Alterations and Their Clinical Implications
Source: J Oral Pathol Med. 2026 Jul 20;55(9):1008–18. doi: 10.1111/jop.70172 (PMC13635271; doi:10.1111/jop.70172)
Supplement: Supplementary file 3 [file JOP-55-1008-s003.docx]

**Table S2. Risk of Bias Assessment – Newcastle–Ottawa Scale (NOS)**

*Applied to case-control studies (n = 12)*

| **Study** | **Selection (max ★★★★)** | **Comparability (max ★★)** | **Exposure (max ★★★)** | **Score** | **Risk of Bias** |
| --- | --- | --- | --- | --- | --- |
| Segura-López et al., 2015 | ★★★ | ★ | ★★ | 6 | **Moderate** |
| Maruyama et al., 2017 | ★★ | ★ | ★★ | 5 | **Moderate** |
| Fan et al., 2018 | ★★★★ | ★★ | ★★★ | 9 | **Low** |
| Lee et al., 2020 | ★★★ | ★★ | ★★ | 7 | **Low** |
| Okuda et al., 2022 | ★★★ | ★★ | ★★ | 7 | **Low** |
| Narayanan et al., 2023 | ★★★ | ★★ | ★★★ | 8 | **Low** |
| Nouri et al., 2023 | ★★ | ★ | ★★ | 5 | **Moderate** |
| Chen et al., 2023 | ★★★★ | ★★ | ★★ | 8 | **Low** |
| McKinley et al., 2023 | ★★★ | ★ | ★★★ | 7 | **Low** |
| McNamara et al., 2025 | ★★★★ | ★★ | ★★★ | 9 | **Low** |
| Uguz et al., 2025 | ★★★★ | ★★ | ★★ | 8 | **Low** |
| Oh et al., 2025 | ★★★★ | ★★ | ★★★ | 9 | **Low** |

*Score interpretation: 1–3 = High risk; 4–6 = Moderate risk; 7–9 = Low risk.*

*★ = criterion met; absence of star = criterion not met or unclear.*
